# Insecticide-treated net (ITN) ownership, usage, and malaria transmission in the highlands of western Kenya

**DOI:** 10.1186/1756-3305-4-113

**Published:** 2011-06-18

**Authors:** Harrysone E Atieli, Guofa Zhou, Yaw Afrane, Ming-Chieh Lee, Isaac Mwanzo, Andrew K Githeko, Guiyun Yan

**Affiliations:** 1 Climate and Human Health Research Unit, Centre for Global Health Research, Kenya Medical Research Institute, P.O. Box 1578-40100, Kisumu, Kenya; 2 Program in Public Health, College of Health Sciences, University of California, Irvine, CA 92697, USA; 3 Public Health Department, School of Health Sciences, Kenyatta University, P.O Box 43844-00100, Nairobi, Kenya

## Abstract

**Background:**

Insecticide-treated bed nets (ITNs) are known to be highly effective in reducing malaria morbidity and mortality. However, usage varies among households, and such variations in actual usage may seriously limit the potential impact of nets and cause spatial heterogeneity on malaria transmission. This study examined ITN ownership and underlying factors for among-household variation in use, and malaria transmission in two highland regions of western Kenya.

**Methods:**

Cross-sectional surveys were conducted on ITN ownership (possession), compliance (actual usage among those who own ITNs), and malaria infections in occupants of randomly sampled houses in the dry and the rainy seasons of 2009.

**Results:**

Despite ITN ownership reaching more than 71%, compliance was low at 56.3%. The compliance rate was significantly higher during the rainy season compared with the dry season (62% vs. 49.6%). Both malaria parasite prevalence (11.8% vs. 5.1%) and vector densities (1.0 vs.0.4 female/house/night) were significantly higher during the rainy season than during the dry season. Other important factors affecting the use of ITNs include: a household education level of at least primary school level, significantly high numbers of nuisance mosquitoes, and low indoor temperatures. Malaria prevalence in the rainy season was about 30% lower in ITN users than in non-ITN users, but this percentage was not significantly different during the dry season.

**Conclusion:**

In malaria hypo-mesoendemic highland regions of western Kenya, the gap between ITNownership and usage is generally high with greater usage recorded during the high transmission season. Because of the low compliance among those who own ITNs, there is a need to sensitize households on sustained use of ITNs in order to optimize their role as a malaria control tool.

## Background

Insecticide-treated mosquito nets (ITNs) used for protection against mosquito bites have proven to be a practical, highly effective, and cost-effective intervention against malaria [1]. The evidence of the public health impact of ITNs, supporting their wide-scale use in Africa, is drawn from areas of stable malaria transmission where *Plasmodium falciparum *infection prevalence in the community is often over 40% [1,2]. Community-based randomized controlled trials (RCT) in these regions have documented average reductions of 20% in all causes of mortality in children under 5 years old within 2 years of increasing ITN use from 0 to 50-70% [3-8]. Scaling up ITN coverage and use by young children and pregnant women has been made a consensus target of the Millennium Development Goals (MDGs), the Roll Back Malaria Partnership (RBM), and the US President's Malaria Initiative (PMI) [9-11]. Targeting individual protection to these vulnerable groups [12-14] is a well-founded and explicitly accepted priority of all three initiatives because these groups bear the highest risk of morbidity and mortality from malaria. However, this strategy ignores the potentially greater community-wide benefits of broader population coverage [15] in areas of moderate and seasonal transmissions, like the highlands, where all age groups are equally vulnerable and no explicit resources, targets, or strategies have been proposed to achieve protection.

Unlike the lowlands where perennial malaria transmission is attributed to high vector densities throughout the year [16], transmission intensity and vector densities in the highlands are relatively low and seasonal (17). Because of this, human populations across age groups have a poorly developed immunity to malaria because exposures are infrequent [17], and they are therefore vulnerable to severe clinical illness and complications from *Plasmodium *infection [18,19]. In addition, research indicates that the mechanisms leading to malaria transmission in the highlands are complex and are probably due to the combined effects of factors such as topography [20], hydrology [21], climate variability [22], land-use/land-cover change [23], and drug resistance [22]. Given these characteristics, ITNs are considered one of the most effective intervention strategies for the control of malaria across all age groups in the highland regions [15].

Although ITN distribution has been massively expanded in most parts of malaria endemic sub-Saharan countries since 2005, there is limited information on community based actual use of nets owned, area specific reasons for non-use, and the possible impact of the variations in use on malaria vector densities and transmission in either Kenyan highlands or other countries where malaria is seasonal and unstable. Most studies of ITN use are intervention trials that attempt to explain why vulnerable groups, such as children under five or pregnant women, are or are not using an ITN [24,25], or describe which household members use the owned ITN(s) [26,27]. Earlier studies on ITN coverage, use, and impact on malaria transmission have different approaches, with most having common conclusions, such as higher ITN use in the high transmission season when mosquito densities are high, and indoor temperatures are low, with the main focus on children under the age of 5 years and pregnant women [8,26,28-30]. As far as could be ascertained, there is inadequate vectorial and parasitological information, or data on the actual use of owned ITNs and their health impacts in areas of Africa that support low or unstable transmission since the inception of the scaling up of ITN coverage. The objective of this study was to examine the utilization of ITNs delivered by the Kenya Ministry of Health (MOH) facilities under routine operational conditions in areas of moderate to unstable/seasonal malaria transmission in two highlands sites in western Kenya.

## Materials and methods

### Study area

**Figure 1:**
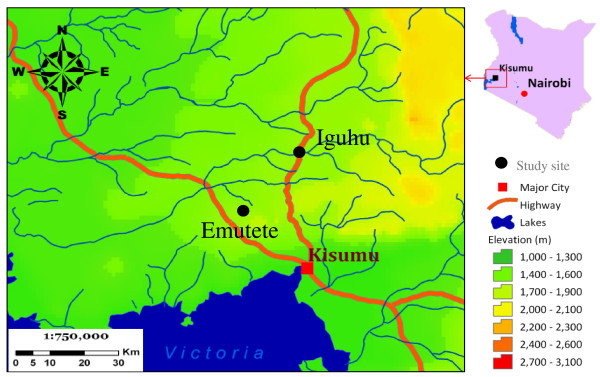
**Study area map showing two study sites: Iguhu in Kakamega district and Emutete in Emuhaya district**.

The two study regions, Iguhu (0°11' N, 34°44' E, 1,430-1,570 m a.s.l) in Kakamega district, and Emutete (0°02' N, 34°38' E, 1,480-1,650 m a.s.l) in Emuhaya district are situated in the highlands of Western Kenya (Figure 1). The average population density was over 900 persons/km^2 ^[31]. The climate in western Kenya generally consists of a bimodal pattern of rainfall, with the long rainy season from April to June and a short rainy season in October and November. There is no clear dry season, but usually there is less rainfall from July to September. January and February are the hottest and the dry months. The epidemiology of malaria in the study areas is defined as epidemic-prone districts (risk class 5-20%). Malaria transmission peaks during the long rainy season.

### Selection of study houses

A total of 600 houses, 300 houses from each of the two sites were randomly selected within an area of approximately 4 × 4 km^2^. The GPS (Global Positioning System) coordinates of each sampled house were taken and then mapped using a handheld GPS unit. Owners of the selected houses were requested to sign a freely administered informed consent form for participation in the study, and for entomologic, parasitological and questionnaire surveys.

### Entomological data collection

Mosquitoes were collected using the Pyrethrum Spray Collection (PSC) method [32] during the dry (February-March 2009, low transmission season) and the rainy (May-June 2009, high transmission season) seasons from all the study houses. Mosquito samples were taken to the Kenya Medical Research Institute (KEMRI) laboratory in Kisumu, for mosquito morphological identification.

### Parasitological data collection

Occupants from sprayed houses who freely consented to participate in the study were screened for the malaria parasite during both the dry and the rainy season in 2009. Rapid diagnostic tests kits (RDTs), ***Paracheck-Pf***^® ^(Orchid Biomedical Systems, Goa, India), were used for field identification of infections, but microscopy was used to determine infection prevalence rates. Thin and thick blood smears were taken in the field and the slides were stained with 4% Giemsa for 30 minutes [33]. Households with absentees were revisited the following day to recruit those missing at the first visit. Participants with positive slide tests and with malaria symptoms were offered free treatment with artemisinin-based combination therapy (ACT) according to Kenya national MOH guidelines [34]. Participants with complicated malaria cases during our survey were advised to visit the nearest health facility and transportation was provided for those who needed help to get to the facility.

### ITN ownership and usage

Households were interviewed about ITN ownership and use as a means of malaria vector control. In general, a household was defined as a unit headed by a male or female with his ⁄ her dependents and spouse, and who share a cooking pot/common eating place and sleep under one roof [35]. An ITN was defined as any long-lasting insecticide treated net (LLIN) or a conventional net treated within the past 12 months [10]. Bed net ownership rate was calculated as the ratio of the number of households with at least one bed net over the total number of households surveyed. Population coverage with bed nets was computed as the ratio of the total number of individuals reporting sleeping under a bed net, regardless of the condition and type of the net, over the total number of individuals surveyed. Bed net usage was defined as the percentage of nets that were actually used the previous night over the total number of ITNs/LLINs surveyed. Other variables that were assumed to determine ITN use such as education level of households, general knowledge of malaria vectors, knowledge of hanging bed nets, household sleeping patterns, and demographics data were also recorded using a questionnaire.

### Data management and analysis

Data was collected and entered in Excel spread sheets (Microsoft Corporation) and statistical analysis was performed by the use of STATA SE 9 (StataCorp LP, College Station, TX USA). The χ^2^-test was used to determine the differences between ITN ownership and actual usage. The Student t-test was used to determine differences in vector densities between houses with at least a functional ITN and those with none in use.

### Ethical consideration

Ethical clearance was obtained from the Ethical Review Committee of Kenya Medical Research Institute, "Ecology of African highland malaria (II), SSC No. 1382 (N)" dated May 15^th ^2008 and the Institutional Review Board of the University of California at Irvine. A freely administered informed consent with interpreters was given to residents for participation in the study.

## Results

### Characteristics of study participants

During the dry and the rainy seasons, a total of 600 houses per season (300 houses from each site) were surveyed. A total of 1,160 and 1,265 participants of all ages were surveyed in the dry and rainy seasons respectively (Table 1). Slightly less than 60% of the study participants, in both the dry and rainy seasons, were from Emutete, and the remainders were from Iguhu (Table 1). As shown in Table 1 there were more female than male participants in both the dry and the rainy season and with age specific variations.

**Table 1:** Characteristics of the study participants by season

	Dry season (*n *= 1160)	Rainy season (*n *= 1265)
**Site**		
Emutete	669 (57.7%)	745 (58.9%)
Iguhu	491 (42.3%)	520 (41.1%)
**Sex**		
Male	522 (45.0%)	520 (41.1%)
Female	638 (55.0%)	745 (58.9%)
**Age groups**		
≤ 4	301 (26.0%)	326 (25.8%)
5-14	381 (32.8%)	437 (34.5%)
≥ 15	478 (41.2%)	502 (39.7%)
**Overall**		
ITN ownership	858 (74.0%)	909 (71.4%)
ITN use	426 (36.6%)	560 (44.3%)
Parasite infection rate	75 (6.5%)	189 (15.0%)

### Household ITN ownership and usage

**Figure 2:**
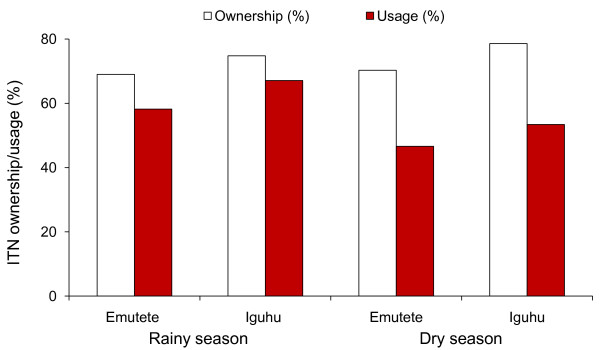
**Seasonality in bednet ownership and usage rate in the two study sites (Iguhu and Emutete)**.

Reported and confirmed net ownership and usage among the study participants is summarized in Figure 2. The overall percentage of participants who owned at least one ITN was 73.8% in the dry season and 71.4% in the rainy season. There was an inter-site difference in ITN ownership (Figure 2). The seasonal ITN usage during the dry season was significantly lower than that in the rainy season (49.5% vs. 61.8%, OR = 0.6, χ ^2 ^= 37.8, d.f. = 1, P < 0.0001). In both regions, participants' ITN usage was significantly higher in the rainy than the dry season (Figure 2). The proportion of non-compliant individuals (those who owned ITNs but did not use them) was significantly higher during the dry season than rainy season, 46.6% vs. 32.9% in Iguhu (χ ^2 ^= 12.42, d.f. = 1, P < 0.001) and 53.4% vs. 41.8% in Emutete (χ ^2 ^= 21.12, d.f. = 1, P < 0.0001), respectively.

**Figure 3:**
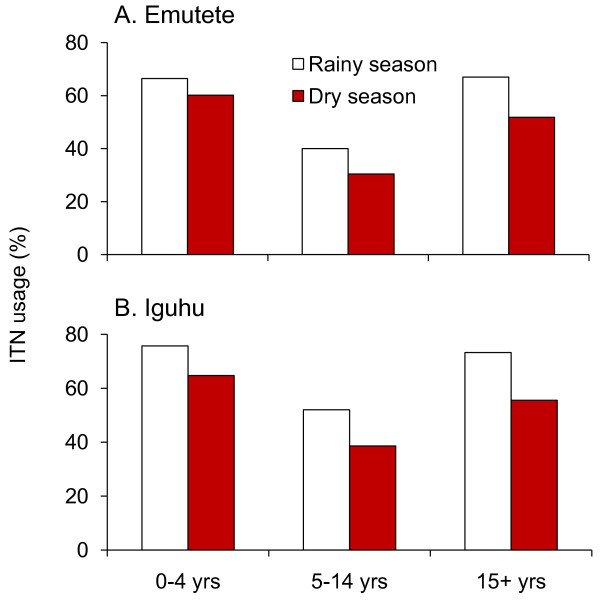
**Age-specific ITN usage**.

Figure 3 shows age specific net usage among those who owned ITNs during the dry and the rainy season. The age specific ITN usage across the age groups was 71.3%, with significantly higher usage for children under 5 years (percent usage of 78.4%) than both the 5-14 year age group (percent usage of 69.1%) and the 15 year and older age group (percent usage of 72.2%).. There was a significant inter-site difference in ITN usage for different age groups (Figure 3). For example, for the ≥ 15 year age group, the ITN usage was 67.9% in Emutete whereas it was 78.5% in Iguhu (χ ^2 ^= 13.28, d.f. = 1, P < 0.001) (Figure 3).

Compared with ownership, actual ITN use was considerably lower, regardless of seasonality and inter-site variation (Figure 3). There was a significant seasonal change in ITN usage in all age groups. For example, the ITN usage for ages ≥ 15 years was 56.4% during the rainy season compared to 43.8% during the dry season (χ ^2 ^= 6.31, d.f. = 1, P = 0.01). There was also a significant inter-site difference in ITN usage for different age groups (Figure 3). For example, the ITN usage for children under 5 years during the dry season was 62.3% in Iguhu but it was 49.2% in Emutete (χ ^2 ^= 5.38, d.f. = 1, P = 0.02). Determination of ITN coverage was a limiting factor as this would only be ascertained by doing spot checks very early in the morning to confirm participants who had their nets properly hung.

### Malaria prevalence and ITN usage

**Figure 4:**
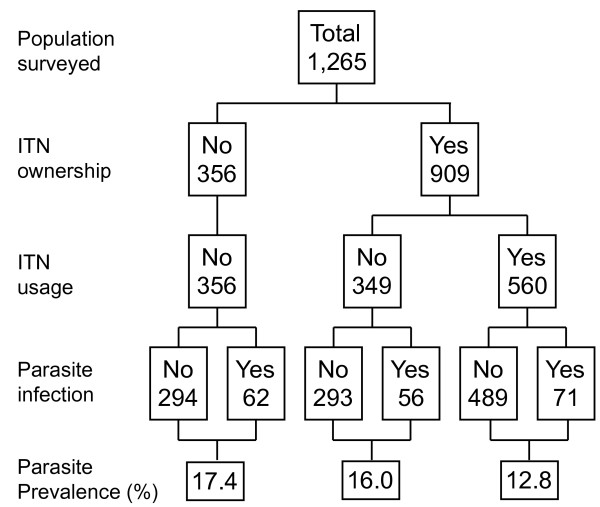
**Bednet use and malaria parasite prevalence during the rainy season**.

**Figure 5:**
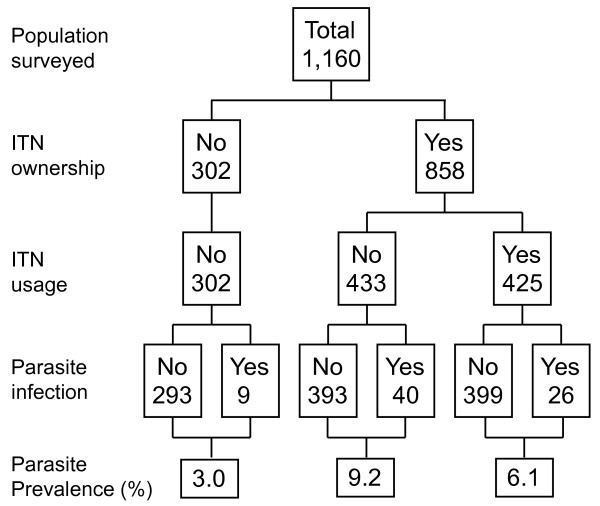
**Bednet use and malaria parasite prevalence during the dry season**.

**Figure 6:**
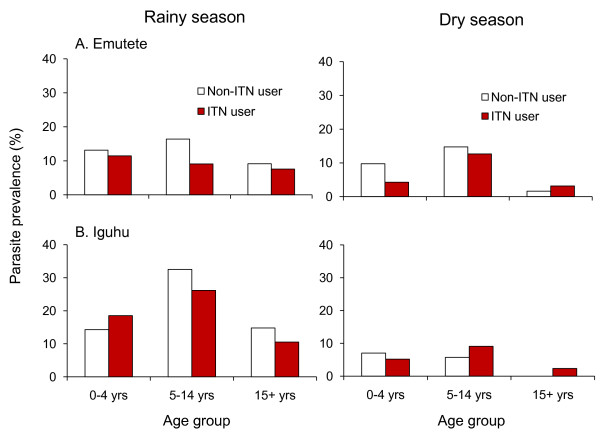
**Age-specific malaria parasite prevalence among ITN and non-ITN users**.

Figures 4 and 5 summarize the prevalence of malaria infection by non-use and use of ITNs during the rainy and dry seasons respectively. Overall infection prevalence for all age groups was significantly lower among net users compared to non-net users (12.8% vs. 16.7%) during the rainy season (OR 0.72, 95% CI 0.51-1.00, P <0.05) (Figure 4). Similarly, during the dry season infection prevalence was lower among net users than non-net users (6.1% vs 6.6%) although they were not statistically different. (Figure 5). Figure 6 illustrates differences in age-specific malaria prevalence between ITN users and non-ITN users across age groups. In Emutete, the parasite infection rate was 9.0% in ITN users compared with 13.0% in the non-ITN user group (P = 0.09), whereas in Iguhu parasite prevalence was 16.9% in ITN users compared with 23.2% in the non-ITN user group (P = 0.07) (Figure 6). During the dry season, the overall infection prevalence was low compared with the rainy season, and it was similar between ITN users and non-users (6.1% vs. 6.7% in Iguhu and 7.3% vs. 8.4% in Emutete). There were significant differences in parasite prevalence among different age groups, with the highest infection rate in the 5-14 year age group. This group also showed the highest reduction in parasite prevalence in ITN users relative to non-ITN users during infection peak season, i.e., the rainy season (Figure 6).

### Malaria vectors density and ITN usage

Due to the very low *An. funestus *densities in the two highlands sites, this data was categorized into season for analysis. Unlike in the dry season where there was no difference in the number of indoor *An. funestus *collected in the houses with or without ITNs, there was significantly high number of *An. funestus *collected in houses without an ITN compared with those with one during the wet season (χ ^2^= 3.86, P = 0.04). Table 2 shows *An. gambiae *s.l. density during the two study seasons. During the dry season, we did not detect significant differences in indoor resting female *An. gambiae *s.l. density between ITN owning houses and houses without ITNs (Table 2). Indoor resting *An. gambiae *s.l. density in Emutete was 0.58 females/house/night (f/h/n) in houses using ITNs compared with 0.80 f/h/n in houses not using ITNs (t = 1.25, d.f. = 282, P = 0.21). *An. gambiae *s.l. density in Iguhu was 0.10 females/house/night (f/h/n) in houses using ITNs compared with 0.09 f/h/n in houses not using ITNs (t = 0.06, d.f. = 262, P = 0.95). However, during the rainy season, indoor resting female *An. gambiae *s.l. density in houses with functional ITNs were significantly lower, 43% lower, compared to those not using ITNs in both Emutete and Iguhu (Table 2). Indoor resting female *An. gambiae *s.l. densities in Iguhu were 0.64 f/h/n and 1.10 f/h/n in houses using and not using ITNs, respectively (t = 1.96, d.f. = 212, P = 0.05), whereas those densities were 0.90 f/h/n and 1.56 f/h/n in Emutete (t = 2.38, d.f. = 167, P = 0.02).

**Table 2:** Household bed net coverage and *Anopheles gambiae s.l *density (female/house/night) during the dry and rainy seasons.

Season	Site	*An. gambiae *density (95% CI)	
		
		Household without ITN	Household with ITN ^+^
Dry season	Emutete	0.80 [0.48, 1.13]	0.58 [0.37, 0.79]
	Iguhu	0.09 [0.02, 0.17]	0.10 [0.00, 0.19]
Rainy season	Emutete	1.58 [1.04, 2.07]	0.90 [0.68, 1.12] *
	Iguhu	1.11 [0.72, 1.50]	0.64 [0.44, 0.84] *

^+ ^Household with ITN is defined as household that has at least one ITN being used at the time of survey.

* Significant difference at level of 0.05 in two sample t-test assuming unequal variances.

### Factors affecting ITN use

Regardless of the types of nets, 86.3% and 94.3% of the nets in Iguhu and Emutete respectively were in good condition, i.e., not torn. In Iguhu 89.8% of the surveyed nets were LLINs (85.1%) or retreated regular ITNs (4.7%), whereas this number is 86.2% in Emutete (62.4% LLINs), the remainders were untreated nets. Overall, about 20% of the surveyed ITN/LLINs were not considered as good for preventing mosquito biting because they had at least one tear.

Education level and knowledge about malaria transmission were some of the significant reasons affecting ownership and usage of ITNs (Table 3). When compared to household with no education, households with at least a member having primary or secondary education level had significant higher (p <0.05) percentage ITN ownership. When asked about malaria transmission knowledge, those houses with at least a member having primary or secondary level of education had knowledge about malaria and with significant high level (p <0.05) of ITN ownership than those with no knowledge. Although there were variations between study sites, in general, houses with non-educated parents or guardians had significantly lower ITN ownership, fewer ITNs, lower ITN usage, and significantly less knowledge about malaria prevention using ITNs (Table 3).

**Table 3:** Education level of household heads or guardians and bed net usage.

		Education level *		
		
Site	ITN usage and other factors	None	Primary	Secondary or above
Emutete	Average number of human adults per house	1.58 ^a^	1.79 ^a^	2.08 ^b^
	Average number of children per house	2.12 ^a^	2.29 ^a^	2.54 ^a^
	Houses with ITNs (%)	43.18 ^a^	69.92 ^b^	68.69 ^b^
	Average number of ITNs per house	0.59 ^a^	0.91 ^b^	1.27 ^c^
	ITN usage in adults in houses owning ITN (%)	79.17 ^a^	75.16 ^a^	72.60 ^a^
	ITN usage in children in houses owning ITN (%)	43.06 ^a^	47.83 ^a^	51.70 ^a^
	ITN usage in adults: overall (%)	36.54 ^a^	53.64 ^b^	51.46 ^b^
	ITN usage in children: overall (%)	22.14 ^a^	35.11 ^b^	36.25 ^b^
	Percentage of people knows that ITN prevents malaria	30.30 ^a^	56.91 ^b^	52.53 ^b^
				
Iguhu	Average number of human adults per house	1.20 ^a^	1.46 ^a^	1.36 ^a^
	Average number of children per house	1.34 ^a^	1.53 ^a^	1.46 ^a^
	Houses with ITNs (%)	40.16 ^a^	65.93 ^b^	67.95 ^b^
	Average number of ITNs per house	0.46 ^a^	0.87 ^b^	1.05 ^b^
	ITN usage in adults in houses owning ITN (%)	87.32 ^a^	94.41 ^a^	91.76 ^a^
	ITN usage in children in houses owning ITN (%)	52.50 ^a^	62.50 ^b^	68.42 ^b^
	ITN usage in adults: overall (%)	40.79 ^a^	68.53 ^b^	73.58 ^b^
	ITN usage in children: overall (%)	24.71 ^a^	46.12 ^b^	57.02 ^b^
	Percentage of people knows that ITN prevents malaria	38.58 ^a^	65.93 ^b ^	67.95 ^b^

* Same letter in the same row indicates no significant difference whereas different letters in the same row represent significant difference at p = 0.05 level.

## Discussion

Most parts of Kenya have approached or met the RBM household ITN coverage target of at least 60% as reported by previous studies [36]. Similarly, after almost six years of MOH efforts towards distribution of nets on a national scale, this study has shown that despite current high net ownership (>60%) within the highlands, actual usage remains low, with only approximately 53% of the residents who own nets reporting net use. This scenario may be a set-back on the intended and expected role and impact of distributed ITNs. As observed, most of the unused nets were either new and kept safely from damage or hung but not used. Low education level of the head of the household is one of the reasons why such a high percentage of the population own but do not use nets. Compared with heads of household with no education at all, the majority of heads of household with at least a primary level education know that mosquitoes transmit malaria and they eventually tend to acquire and use nets. Another reason mentioned by majority respondents was seasonality, with the onset of the colder/rainy season improving use of nets among those who own them, either because of the cold weather or because of noted presence of biting mosquitoes. This observation is consistent with previous studies conducted earlier in Ethiopia, where 35% of nets owned were not being used [37]. In contrast, studies in Sierra Leone and Madagascar recorded very low percentages of owned but unused nets (5.1% and 5.6% respectively) [38]. There is therefore need for targeted sensitization of the less educated group on importance of ITN ownership and usage so as to scale-up utilization. Likewise, there is need to sensitize the community on the importance of ITN adherence throughout the seasons with emphasis that malaria transmission occurs year round thus need for protection regularly. One of the probable ways to approach this may be through routine household visit by trained community health workers.

Even though ITN ownership was similarly high across seasons, seasonality in use was remarkable during this survey in both regions of study. Net use was considerably higher in the rainy, cooler season of April-May than in the dry, hot season of January-February. During the rainy season, the non-compliance rate was lower compared with the dry season. Similar findings have also been reported in surveys in ITN intervention trials in the lowlands of western Kenya and northern Ghana [26,39]. As in this highland region, the rainy months are characterized by the highest incidence of mosquito biting and malaria transmission. Unlike most parts of Africa where malaria is perennial and not limited to the rainy season [40], this area experiences seasonality in malaria transmission, but with "stable" transmission in areas along the valley bottoms [22]. Because of stable vector breeding habitats in the valley bottoms in the highlands [41], malaria transmission occurs year round, but with low intensity, and thus the need for continuous use of ITNs. Lower use of ITNs during the dry season with the assumption of no vectors and therefore no transmission can be detrimental as experienced in Ouagadougou in Burkina Faso, where most malaria occurs in the hotter months just after the rains [42]. Remarkable seasonality in net use reported in this area highlights the need for education to promote year-round use. For accurate monitoring and evaluation of ITN impact on malaria transmission, it would be preferable to conduct daily surveys on household ITN use year round to capture both low and high malaria transmission seasons. Unfortunately, this study could not accomplish this because of logistic difficulties. However, this study was able to undertake a two season survey in two similar geographic highland regions. This made it more appropriate to compare between surveys conducted in different seasons with less bias.

The proportion of households possessing mosquito net(s) and the proportion of children less than 5 years of age who slept under a net the preceding night are two of the key RBM bed net ownership indicators used to investigate the strengths and weaknesses of monitoring malaria control [10]. In this study, the comparative difference in use by children and adults did not depend on any characteristic of the surveys, such as region or season. Usage of young children below age of 5 years with nets was often as high as or higher than for adults above 15 years old. Children between ages of 5-14 years had significantly lower usage during the dry and rainy seasons in both the regions. This phenomenon has also been found elsewhere in studies in Uganda [43], in Ghana and The Gambia, but in contrast with earlier findings from, Kenya, Rwanda, Zimbabwe and Burkina Faso, where the pattern was inverse [24], with adult ITN use being higher than that for children under 5 years of age. Another contrasting finding was observed in rural south central Somalia, where net use in younger children, older children, and adults was not different [44]. It thus appears that young children are not at a disadvantage in the allocation of scarce nets and eventual use within households in either of the regions in any season.

The protective effect of nets on infection prevalence during the rainy season among sampled participants was consistent across age-groups except for ages under 5 years in Iguhu. Unlike in the rainy season, the nets' protection role was not clearly observed in older children and adults in the dry season. Children under 5 years old in both regions had the largest protection margin with net use. Overall, during the dry season infection prevalence among net users was lower than in non-users by 56.7% in Emutete and by 26.2% in Iguhu among this age group. During this season, the odds of malaria infection among this age group were 2.3 fold and 1.4 fold higher among non-net users in Emutete and Iguhu respectively. While the approach of targeting interventions to protect at-risk individuals is based on solid scientific grounds [14,45,46], and is widely accepted [12], this strategy should not preclude efforts to maximize communal protection through less selective delivery mechanisms, more so in areas where all age groups are vulnerable. Targeting limited subsidies to maximize personal protection of the most vulnerable should remain a priority, mostly in malaria endemic zones, but more equitable and effective suppression of risk for entire populations in hypo-meso endemic areas where all ages are more susceptible to malaria infections should be encouraged. Likewise, given the relatively higher prevalence of infection through older childhood and into adulthood, during high transmission seasons, it is important to recognize the need to provide ITNs to all members of a community, and not to focus only on young children in areas of low transmission. This resonates with recent calls for high coverage among all community members across the range of transmission settings [47] where it is also recognized that individuals older than five years contribute to transmission.

The impacts of ITNs very much depend on their excito-repellent and insecticidal properties [48,49]. Furthermore, the interaction of these two properties, to yield maximum levels of personal and communal protection, is complex and has crucial implications for ITN programmes across Africa [29]. Households with a functional net during high transmission season, when densities of malaria vectors are high had significantly fewer mosquitoes than those without a functional net. These household vector reduction outcomes concur with findings from earlier studies on the properties of ITNs where it has been clearly shown that ITNs reduce malaria risk among unprotected individuals by suppressing the density, survival [50], and feeding frequency [51] of malaria vector populations. With both insecticidal and excito-repellent properties, ITNs can protect not only the individuals and households that use them, but also members of the surrounding community [15]. This is because they kill adult mosquitoes directly or force them to undertake longer, more hazardous foraging expeditions in search of vertebrate blood and aquatic habits. They repel mosquitoes to reduce the frequency with which they successfully acquire blood, often diverting them to feed on other mammals that do not host the malaria parasite, resulting in greatly reduced prevalence of sporozoite infection [29]. These two major properties add to the effectiveness of ITNs for personal protection because they constitute the major motivating force behind ITN uptake and use at the individual and subsequently the community level.

## Conclusion

This study demonstrates that in hypo-meso endemic highlands of western Kenya, despite high mosquito net ownership, actual usage is still remarkably low. To track progress and draw inferences about this principal malaria intervention tool, both ownership and actual use indicators must be taken into account. Regular, region-specific rapid assessments of household possession, use of nets, and respondent's knowledge should complement ongoing distribution, and findings should be incorporated into programme policy. Based on these findings, ITN use in this region could be increased, if IEC messages on malaria transmission were strengthened either through community health workers or net provider programs to encourage participants to use nets they already possess. Since the analyses found that there were disparities in net use across age groups, distribution of free or subsidized nets to the entire population may optimize coverage as well as help increase ITN use.

## Competing interests

The authors declare that they have no competing interests.

## Authors' contributions

HA carried out the field surveys, assembled data, analyzed and drafted the manuscript. GZ performed statistical analysis and revised the manuscript. MCL participated in writing and revised the manuscript, YA and IM participated in study coordination and revised the manuscript. AG and GY designed the study and helped with manuscript preparation. All authors read and approved the final manuscript.
